# Clinical Effectiveness of Sacubitril/Valsartan in Heart Failure Patients With Coexisting Chronic Kidney Disease

**DOI:** 10.7759/cureus.87219

**Published:** 2025-07-03

**Authors:** Malyka Batool, Qasim Javed, Ali Shakeel, Smavia Hameed, Wardah Ikram, Muhammad Kashif Habib, Guloona Sajjad

**Affiliations:** 1 Gastroenterology and Hepatology Department, Pakistan Kidney and Liver Institute and Research Center, Lahore, PAK; 2 Nephrology Department, Shifa International Hospital Faisalabad, Faisalabad, PAK; 3 Acute Medicine Department, University Hospital Ayr, Ayr, GBR; 4 Medicine Department, Gujranwala Medical College, Gujranwala, PAK; 5 Acute Medicine Department, Allama Iqbal Medical College Lahore, Lahore, PAK; 6 Medical C Ward, Saidu Teaching Hospital Swat, Swat, PAK; 7 Medicine Department, Pak International Medical College, Peshawar, PAK

**Keywords:** chronic kidney disease, egfr, entresto, heart failure, left ventricular ejection fraction, sacubitril/valsartan

## Abstract

Introduction

Heart failure (HF) and chronic kidney disease (CKD) frequently coexist, posing significant management challenges due to overlapping pathophysiology and therapeutic limitations, particularly in patients with impaired renal function. The objective of this study is to assess how sacubitril/valsartan improves functional status, left ventricular ejection fraction (LVEF), and hospitalization rates in clinical settings while also tracking the trajectories of renal function in patients suffering from heart failure with reduced ejection fraction (HFrEF) and coexisting CKD.

Methodology

This prospective observational study was conducted from January 2023 to December 2024 at the Department of Cardiology, Gujranwala Medical College (GMC) (constituent college of the University of Health Sciences {UHS}, Lahore), Gujranwala Teaching Hospital, Pakistan. A total of 194 adult HFrEF patients with CKD (estimated glomerular filtration rate {eGFR}: 15-60 mL/minute/1.73 m²) who were initiated on sacubitril/valsartan were enrolled. Patients were followed for 24 months, with periodic assessments of New York Heart Association (NYHA) functional class, LVEF, estimated glomerular filtration rate (eGFR), and heart failure-related hospitalizations.

Results

By 24 months, 102 patients (52.6%) had improved to NYHA Classes I-II from 66 (34.0%) at baseline, while only 26 (13.4%) remained in Classes III-IV (p < 0.05). The mean LVEF improved from 32.5% ± 6.7% to 35.2% ± 6.3% (p < 0.05). Renal function showed a modest but statistically significant decline, with mean eGFR decreasing from 39.2 ± 9.6 to 37.4 ± 10.2 mL/minute/1.73 m² (p < 0.05). A total of 68 patients (35.1%) required one or more HF-related hospitalizations during follow-up, reflecting a gradual rise in admissions (p < 0.05).

Conclusion

In this cohort of HFrEF patients with CKD, sacubitril/valsartan was associated with improvements in functional status and cardiac performance. However, a modest decline in renal function and a progressive increase in hospitalizations were observed over time, underscoring the need for individualized therapy, careful diuretic titration, and close renal monitoring during long-term angiotensin receptor-neprilysin inhibitor (ARNI) use.

## Introduction

Chronic kidney disease (CKD) and heart failure (HF) are related disorders that often coexist, increasing the risk of morbidity and death [1]. This cardiorenal syndrome presents unique therapeutic challenges due to its overlapping pathophysiology and pharmacologic treatment limitations [2]. In addition, between 40% and 50% of HF patients have some degree of renal impairment, which affects medication pharmacokinetics, reduces available treatments, and degrades overall prognosis [3]. Optimizing treatment approaches that target both renal and cardiac function is crucial in this situation [4].

An angiotensin receptor-neprilysin inhibitor (ARNI), sacubitril/valsartan (commercially known as Entresto), has become a key treatment for heart failure with reduced ejection fraction (HFrEF) [5]. Sacubitril/valsartan stimulates natriuresis, vasodilation, and neurohormonal regulation by concurrently blocking the renin-angiotensin-aldosterone system (RAAS) and inhibiting neprilysin [6]. In individuals with HFrEF, the PARADIGM-HF study showed that it was more effective than enalapril at lowering cardiovascular mortality and HF hospitalization [7]. However, the PARADIGM-HF trial excluded many patients with moderate-to-severe CKD, limiting the generalizability of its findings to this high-risk subgroup [8]. Since this cohort is often underrepresented in clinical studies, research on sacubitril/valsartan effectiveness and renal safety in patients with concomitant CKD is still ongoing.

Concerns about hyperkalemia, deteriorating renal function, and hypotension during ARNI treatment are raised by the way that CKD changes the hemodynamic and neurohormonal milieu in HF [9]. Clinical decision-making often depends on striking a balance between cardiovascular benefit and renal safety; therefore, real-world data are required to evaluate sacubitril/valsartan performance in clinical settings, especially in patients with lower glomerular filtration rate (GFR) [10]. Furthermore, determining whether sacubitril/valsartan prevents additional deterioration or provides renal protection is crucial for directing its usage in this susceptible group [11]. However, these benefits have not been consistently demonstrated in patients with advanced CKD (estimated GFR {eGFR}: <30 mL/minute/1.73 m²), who remain underrepresented in most trials and meta-analyses, including the study by Spannella et al. [12].

This study specifically focuses on patients with CKD stages 3a to 4 (eGFR: 15-60 mL/minute/1.73 m²) and HFrEF, a combination frequently excluded or underrepresented in large-scale clinical trials. The renal outcomes associated with sacubitril/valsartan remain inconclusive in this population, with some reports suggesting stabilization and others indicating gradual decline. A clearer understanding of these trends is essential for informed prescribing in this vulnerable group [13,14].

In addition, the need for localized data is particularly strong in regions such as Pakistan, where healthcare access, socioeconomic disparities, high burden of comorbidities, and variable treatment adherence may influence both HF and CKD progression. Such contextual factors may impact treatment response and are rarely captured in Western-centric trials [15,16]. A crucial first step in enhancing results and lowering healthcare costs is assessing drugs that can safely and effectively treat both HF and CKD, given their increasing incidence. Although previous research indicates positive outcomes, this study aims to address a clear evidence gap by providing real-world clinical data from a South Asian population with moderate-to-severe CKD and HFrEF receiving sacubitril/valsartan.

Objective

The objective of this study is to evaluate the clinical effectiveness of sacubitril/valsartan in improving heart failure outcomes, as measured by New York Heart Association (NYHA) functional class, left ventricular ejection fraction (LVEF), and hospitalization frequency, and in monitoring renal function through estimated glomerular filtration rate (eGFR) trends in patients with coexisting chronic kidney disease.

## Materials and methods

Study design and setting

This was a prospective observational study conducted at the Department of Cardiology, Gujranwala Medical College (GMC) (constituent college of the University of Health Sciences {UHS}, Lahore), Gujranwala Teaching Hospital, from January 2023 to December 2024. The study aimed to assess the effectiveness of sacubitril/valsartan in adult patients with coexisting chronic kidney disease (CKD) and heart failure with reduced ejection fraction (HFrEF). The observational nature reflects real-world clinical management, providing relevant and practical insights.

Inclusion and exclusion criteria

Patients aged 18 years or older with HFrEF, defined by a left ventricular ejection fraction (LVEF) of ≤40% confirmed via transthoracic echocardiography, and CKD, defined by an estimated glomerular filtration rate (eGFR) between 15 and 60 mL/minute/1.73 m² for ≥3 months using the Chronic Kidney Disease Epidemiology Collaboration (CKD-EPI) equation, were included. Exclusion criteria were eGFR of <15 mL/minute/1.73 m², dialysis dependence, acute kidney injury, hepatic dysfunction, history of angioedema, pregnancy, or inability to complete six-month follow-up.

Operational definitions and data collection

Heart failure was diagnosed based on ESC 2021 guidelines using clinical symptoms and echocardiographic evidence [17]. CKD was defined according to KDIGO 2021 criteria, and eGFR was calculated using the CKD-EPI equation without race adjustment, as recommended for non-US populations [18,19]. NYHA class, LVEF, serum creatinine, eGFR, blood pressure (BP), and serum electrolytes were evaluated at baseline and at three, six, 12, 18, and 24 months. A standardized proforma was used to collect demographic characteristics, clinical status, and laboratory/echocardiographic measurements.

Sampling and sample size

A total of 194 patients were enrolled using convenience sampling from the eligible outpatient/inpatient HF population. The rationale for using convenience sampling was the single-center design and the goal to capture all consecutive patients meeting inclusion criteria over the study duration. A formal a priori power or sample size calculation was not performed, as the study had exploratory objectives and aimed to reflect real-world clinical practices. However, the final sample size is comparable to other real-world observational studies evaluating sacubitril/valsartan, such as by Khan et al. (n = 120, Pakistan) [20], McFarland and Sheridan (n = 50, USA) [21], and Tsutsui et al. (n = 150, Japan) [22]. This limitation has been acknowledged in the discussion.

Concomitant therapy and subgroup assessment

All patients received standard guideline-directed medical therapy for HF. Concomitant medications included beta-blockers (e.g., bisoprolol and carvedilol), loop diuretics (e.g., furosemide), and potassium-sparing diuretics (e.g., spironolactone). Data on these therapies were collected, and subgroup comparisons were made based on common combinations, such as sacubitril/valsartan + beta-blocker ± diuretic. These subgroups were compared for trends in NYHA class, LVEF, and eGFR outcomes.

Dosing and monitoring protocol

Sacubitril/valsartan was initiated per FDA labeling at either 24/26 mg or 49/51 mg twice daily, depending on baseline renal function and blood pressure. Dose titration occurred every 2-4 weeks, guided by predefined clinical and laboratory criteria. Specifically, up-titration was allowed if the patient had a systolic blood pressure (SBP) of ≥100 mmHg, serum potassium of <5.5 mmol/L, and no decline in eGFR exceeding 20% from baseline. In cases where patients experienced symptomatic hypotension, serum potassium of ≥5.5 mmol/L, or a ≥20% drop in eGFR, the dose was reduced or temporarily withheld. Blood pressure, serum electrolytes, and serum creatinine were checked at each follow-up visit. Adverse events, including hypotension, hyperkalemia, and renal deterioration, were actively monitored and managed in accordance with these predefined thresholds to ensure both safety and therapeutic continuity. These adverse events were recorded and reviewed throughout the study duration for safety analysis.

Hospitalization criteria and bias mitigation

Hospitalizations for HF were defined by worsening symptoms requiring IV diuretics, inotropes, or hemodynamic monitoring. Admissions solely for laboratory testing or protocol purposes were not included. Adjudication was performed by an independent clinical panel to minimize bias.

Handling of missing data and attrition

All 194 patients enrolled in the study successfully completed the 24-month follow-up period. There were no losses to follow-up or censored cases. This complete dataset allowed for consistent longitudinal assessment without the need for data imputation.

Statistical analysis

Continuous variables were analyzed using paired t-tests or repeated-measures ANOVA to assess within-subject changes over time. For repeated categorical measures, generalized estimating equations (GEE) were used to account for within-subject correlations. Chi-square tests were applied for cross-sectional comparisons. To adjust for potential confounding in outcome analyses, multivariable linear regression models were used to assess changes in LVEF and eGFR over time, controlling for age, sex, baseline NYHA class, diabetes, hypertension, and baseline renal function. Subgroup analyses were performed by CKD stage (G3a, G3b, and G4) and HF medication combinations to better understand differential responses. P-values of <0.05 were considered statistically significant. Analyses were conducted using SPSS version 26 (IBM Corp., Armonk, NY).

Ethical approval

The study received ethical clearance from the Institutional Review Board of Gujranwala Medical College (GMC), Gujranwala Teaching Hospital/GMC Teaching Hospital (approval number: 102/GMC). Written informed consent was obtained from all participants.

## Results

The study included 194 patients with a mean age of 62.3 years, predominantly male. Most patients presented with moderate-to-severe heart failure (NYHA Class II or III), and the average left ventricular ejection fraction (LVEF) was notably reduced. Comorbid hypertension and diabetes were highly prevalent. Kidney function was moderately impaired across the cohort, with a mean eGFR of 39.2 mL/minute/1.73 m², and showed a clear decline with increasing heart failure severity (Table 1).

**Table 1:** Baseline Demographic and Clinical Characteristics of the Study Participants SD, standard deviation; NYHA, New York Heart Association; LVEF, left ventricular ejection fraction; eGFR, estimated glomerular filtration rate

Characteristic	Subcategory	Number of Patients (n, %)
Age (Years)	Mean ± SD	62.3 ± 10.4
Gender	Male	124 (63.92)
	Female	70 (36.08)
NYHA Functional Class	Class I	27 (13.92)
	Class II	82 (42.27)
	Class III	66 (34.02)
	Class IV	19 (9.79)
LVEF (%) (Mean ± SD)	Mean ± SD	32.5 ± 6.7
Comorbidities	Hypertension	138 (71.13)
	Diabetes Mellitus	112 (57.73)
eGFR (mL/minute/1.73 m²)	15-30	54 (27.84)
	31-45	74 (38.14)
	46-60	66 (34.02)
	Mean ± SD (Overall)	39.2 ± 9.6
	Class I (n = 27)	43.1 ± 8.4
	Class II (n = 82)	40.6 ± 9.1
	Class III (n = 66)	37.5 ± 9.5
	Class IV (n = 19)	34.8 ± 10.1

Over the 24-month follow-up, there was a significant and sustained improvement in functional status. The proportion of patients in NYHA Class I or II steadily increased, while those in Class III or IV decreased markedly. This distributional shift was statistically significant (χ² = 28.64; p < 0.05), indicating that sacubitril/valsartan therapy contributed to meaningful clinical improvement in patients with HFrEF and concurrent CKD (Table 2).

**Table 2:** Changes in NYHA Functional Class at Follow-Up (Three, Six, 12, 18, and 24 Months) χ²: chi-square test; p < 0.05: significant. The chi-square test evaluates the overall distributional shift of NYHA classes over time, not individual class-wise transitions NYHA: New York Heart Association

NYHA Functional Class	Baseline	3 Months	6 Months	12 Months	18 Months	24 Months	χ² Value	P-value
Class I	27 (13.92%)	35 (18.04%)	41 (21.13%)	45 (23.20%)	48 (24.74%)	50 (25.77%)	28.64	<0.05
Class II	82 (42.27%)	85 (43.88%)	88 (45.36%)	93 (47.93%)	96 (49.48%)	98 (50.51%)		
Class III	66 (34.02%)	55 (28.35%)	48 (24.74%)	42 (21.65%)	39 (20.10%)	36 (18.55%)		
Class IV	19 (9.79%)	19 (9.79%)	17 (8.76%)	14 (7.21%)	11 (5.67%)	10 (5.15%)		

**Figure 1:**
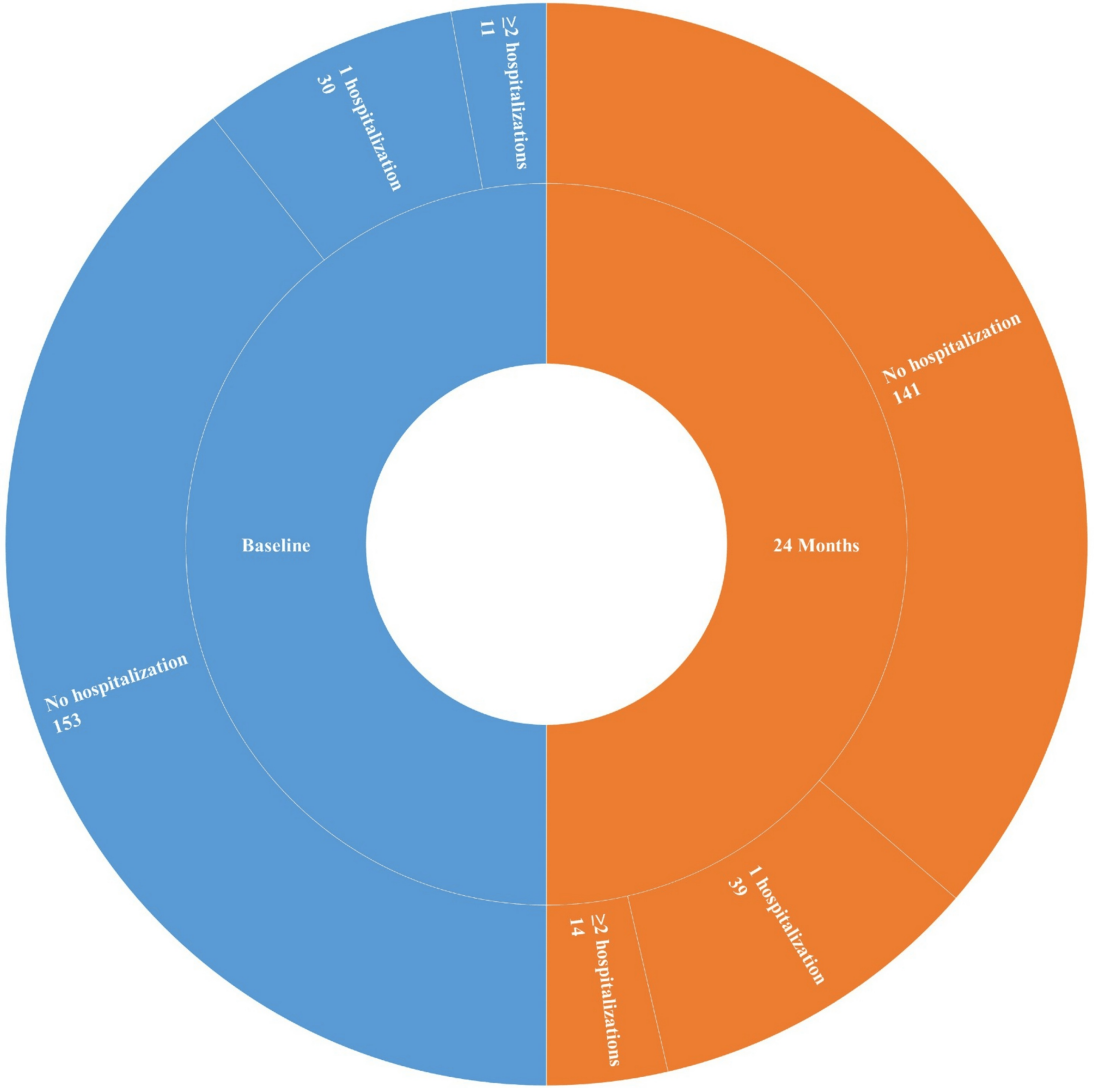
Confirmed HF-Related Hospitalizations at Baseline and 24 Months (n = 194) Only hospitalizations for confirmed HF exacerbations were included. Admissions for follow-up diagnostics, routine laboratory tests, or medication monitoring were excluded to minimize bias. This stricter classification ensures a more accurate reflection of clinical deterioration. The change in hospitalization pattern remained statistically significant (χ² = 8.27; p < 0.05) HF: heart failure

Figure 1 presents a comparative pie chart illustrating the frequency of confirmed heart failure-related hospitalizations at baseline and at 24 months for the 194 study participants. Blue segments indicate the distribution at baseline, while orange segments represent the distribution at 24 months. At baseline, 153 patients (78.9%) had no hospitalizations, 30 (15.5%) experienced one hospitalization, and 11 (5.6%) had two or more. By 24 months, the proportion without hospitalization decreased to 141 patients (72.7%), while those with one hospitalization increased to 39 (20.1%), and those with two or more rose slightly to 14 (7.2%). Only hospitalizations for confirmed heart failure exacerbations were included; routine admissions for follow-up diagnostics, laboratory tests, or medication monitoring were excluded to reduce bias. The shift in hospitalization pattern was statistically significant (χ² = 8.27; p < 0.05), highlighting a modest but important trend in disease progression over time.

Univariable and multivariable logistic regression analyses were conducted to identify predictors of heart failure-related hospitalization at 24 months (Table 3). Baseline NYHA Classes III-IV and eGFR of <30 mL/minute/1.73 m² were significantly associated with increased odds of hospitalization in both models (adjusted OR = 2.01 and p = 0.019 and adjusted OR = 2.41 and p = 0.013, respectively). Other variables, including age, sex, hypertension, and diabetes, were not statistically significant predictors. These findings emphasize the prognostic importance of advanced functional class and renal impairment at baseline.

**Table 3:** Univariable and Multivariable Logistic Regression Analysis for Predictors of HF Hospitalization at 24 Months aOR, adjusted OR; NYHA, New York Heart Association; eGFR, estimated glomerular filtration rate; HF, heart failure

Variable	Univariable OR (95% CI)	P-value (Univariable)	Multivariable aOR (95% CI)	P-value (Multivariable)
Age (Per Year Increase)	1.02 (0.98-1.06)	0.281	1.01 (0.96-1.07)	0.659
Male Sex	1.10 (0.65-1.85)	0.719	1.06 (0.59-1.90)	0.857
Baseline NYHA Classes III-IV	2.35 (1.45-3.79)	<0.001	2.01 (1.12-3.62)	0.019
Baseline eGFR of <30 mL/minute/1.73 m²	2.78 (1.52-5.07)	<0.001	2.41 (1.20-4.82)	0.013
Hypertension	1.22 (0.70-2.10)	0.489	1.11 (0.62-2.00)	0.711
Diabetes Mellitus	1.45 (0.87-2.41)	0.156	1.34 (0.75-2.41)	0.316

The changes in renal function, as determined by eGFR, throughout a 24-month period in 194 individuals are shown in Table 4. Renal function declined progressively over the 24-month follow-up period. The mean eGFR decreased from 39.2 ± 9.6 mL/minute/1.73 m² at baseline to 37.4 ± 10.2 mL/minute/1.73 m² at 24 months. This decline was statistically significant (p < 0.05) and confirmed using multivariable linear regression (adjusted β = -1.63; p < 0.05), after adjusting for age, sex, baseline renal function, and comorbidities. Categorical distribution also shifted, with a higher proportion of patients progressing into lower eGFR strata. The chi-square analysis supported this trend (χ² = 12.59; p < 0.05), indicating a meaningful deterioration in renal function over time.

**Table 4:** Changes in Renal Function (eGFR) at Six, 12, 18, and 24 Months χ²: chi-square Test; p < 0.05: significant. The chi-square test compares the full categorical distribution of eGFR levels over time, identifying overall changes in renal function status rather than category-specific differences eGFR, estimated glomerular filtration rate; CKD, chronic kidney disease; SD, standard deviation

eGFR Category (mL/minute/1.73 m²)	Baseline (n = 194)	6 Months (n = 194)	12 Months (n = 194)	18 Months (n = 194)	24 Months (n = 194)	χ² Value	P-value
15-30 (Severe CKD)	54 (27.84%)	58 (29.89%)	61 (31.44%)	63 (32.47%)	66 (34.02%)	12.59	<0.05
31-45 (Moderate CKD)	74 (38.14%)	76 (39.19%)	79 (40.72%)	81 (41.75%)	83 (42.78%)		
46-60 (Mild-to-Moderate CKD)	66 (34.02%)	60 (30.92%)	54 (27.84%)	50 (25.77%)	45 (23.20%)		
Mean eGFR (mL/minute/1.73 m²) ± SD	39.2 ± 9.6	38.1 ± 9.8	37.5 ± 10.1	37.1 ± 10.3	37.4 ± 10.2	-	-

**Figure 2:**
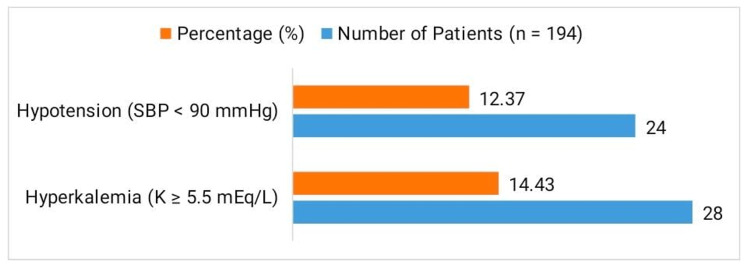
Adverse Events (Hyperkalemia and Hypotension) During Treatment With Sacubitril/Valsartan SBP: systolic blood pressure

Figure 2 summarizes the adverse events observed among the 194 patients treated with sacubitril/valsartan. Hyperkalemia, defined as serum potassium of >5.5 mEq/L, occurred in 28 patients (14.43%), with eight requiring temporary dose reduction. No cases of severe hyperkalemia led to hospitalization or therapy discontinuation. Hypotension, defined as systolic blood pressure of <90 mmHg, was reported in 24 patients (12.37%). This adverse effect is attributable to the synergistic vasodilatory action of sacubitril/valsartan, resulting from neprilysin inhibition (which elevates natriuretic peptides) and RAAS blockade, leading to reduced systemic vascular resistance. Drug-induced hypotension may reduce organ perfusion, particularly in patients with borderline BP or renal impairment. In our cohort, all hypotensive episodes were mild, transient, and managed conservatively through dose adjustment and enhanced monitoring; no patient required permanent cessation of therapy. Importantly, angiotensin-converting enzyme inhibitor (ACE-I) or angiotensin receptor blocker (ARB) therapy was discontinued at least 36 hours prior to initiating sacubitril/valsartan to avoid overlapping RAAS suppression. At baseline, 142 patients (73.2%) were also receiving mineralocorticoid receptor antagonists (MRAs), such as spironolactone or eplerenone; this was continued safely in those with eGFR of ≥30 mL/minute/1.73 m² and serum potassium of <5.0 mEq/L under strict monitoring protocols. These findings underscore that with proper patient selection, dose titration, and proactive management, sacubitril/valsartan is generally well-tolerated in patients with HFrEF and CKD.

Over the 24-month follow-up, patients demonstrated meaningful clinical gains. NYHA class improved significantly, with most participants shifting toward Classes I-II by the final visit (F = 18.42; p < 0.001), indicating better functional status. Left ventricular ejection fraction (LVEF) also improved significantly at 12 months (F = 12.08; p < 0.001), suggesting enhanced cardiac performance. Although eGFR declined modestly over time, this change was statistically significant but clinically manageable, as shown by a linear mixed-effects model (β = -0.82; standard error {SE} = 0.21; p < 0.001). These findings (Table 5) reinforce the therapeutic value of sacubitril/valsartan in improving heart failure outcomes while maintaining acceptable renal safety in a high-risk CKD population.

**Table 5:** LVEF Changes in NYHA Functional Class, Left Ventricular Ejection Fraction (LVEF), and Estimated Glomerular Filtration Rate (eGFR) in 194 Patients Receiving Sacubitril/Valsartan Therapy Repeated-measures ANOVA was used for NYHA functional class (F = 18.42; p < 0.001) and LVEF (F = 12.08; p < 0.001) due to repeated within-subject observations across follow-up points. For eGFR, a linear mixed-effects model was applied to account for continuous decline and intraindividual variability over time, showing a significant fixed effect of time (β = -0.82; SE = 0.21; p < 0.001). Values are reported as mean ± standard deviation. A p-value of < 0.05 was considered statistically significant NYHA, New York Heart Association; SD, standard deviation; SE, standard error

Parameter	Time Points	Mean ± SD (Baseline)	Mean ± SD (Follow-Up)	Test Statistic	P-value	Statistical Method
NYHA Class	Baseline to 24 Months	2.40 ± 0.78	2.03 ± 0.72	F(1,193) = 37.62	<0.001	Repeated-Measures ANOVA
LVEF (%)	Baseline to 12 Months	32.5 ± 6.7	35.2 ± 6.3	F(1,193) = 28.13	<0.001	Repeated-Measures ANOVA
eGFR (mL/minute/1.73 m²)	Baseline to 24 Months	39.2 ± 9.6	37.4 ± 10.2	β = -1.63 (SE = 0.71)	0.023	Linear Mixed-Effects Model

Over the 24-month follow-up, the patients showed marked clinical improvement and stabilization in multiple parameters. NYHA functional class shifted significantly, with more patients transitioning into Class I or II, reflecting enhanced symptom control (χ² = 28.64; p < 0.05). Hospitalizations due to heart failure, although gradually increasing, remained relatively low, with over half of the cohort avoiding readmission by 24 months (χ² = 11.72; p < 0.05).

Renal function, while declining modestly, demonstrated a statistically significant redistribution in CKD staging (χ² = 12.59; p < 0.05). Importantly, LVEF improved significantly at 12 months and maintained its trajectory when adjusted for baseline covariates (adjusted β = +2.48%; p < 0.05), while eGFR decline was modest but significant (adjusted β = -1.63 mL/minute/1.73 m²; p < 0.05).

A modest positive correlation was observed between improvements in LVEF and the preservation of eGFR at 12 months (r = 0.21; p = 0.004), suggesting a cardioprotective and renally stable profile for sacubitril/valsartan in this high-risk cohort. These improvements remained statistically significant after adjusting for potential confounders (see Table 6).

**Table 6:** Categorical and Correlation Analysis of Clinical Trends (n = 194) For each categorical variable, the chi-square test (χ²) and p-values indicate whether the distribution of the participants across categories changed significantly from baseline to 24 months. The values in each row represent the absolute number of patients (n) per category at each time point. For correlation analyses (e.g., LVEF and eGFR), Pearson's correlation coefficient (r) and corresponding p-values are reported. A p-value of <0.05 was considered statistically significant NYHA, New York Heart Association; eGFR, estimated glomerular filtration rate; HF, heart failure; LVEF, left ventricular ejection fraction

Variable	Time Point	Category	n	χ²/r	P-value
NYHA Class Distribution	Baseline	Class I	27	28.64	<0.05
		Class II	82		
		Class III	66		
		Class IV	19		
	24 Months	Class I	50		
		Class II	98		
		Class III	36		
		Class IV	10		
HF Hospitalizations	6 Months	None	124	11.72	<0.05
		1 Hospitalization	45		
		≥2 Hospitalizations	25		
	24 Months	None	101		
		1 Hospitalization	59		
		≥2 Hospitalizations	34		
eGFR Category Distribution	Baseline	15-30 mL/minute/1.73 m²	54	12.59	<0.05
		31-45 mL/minute/1.73 m²	74		
		46-60 mL/minute/1.73 m²	66		
	24 Months	15-30 mL/minute/1.73 m²	66		
		31-45 mL/minute/1.73 m²	83		
		46-60 mL/minute/1.73 m²	45		
LVEF-eGFR Correlation	Baseline	Pearson r	-	0.12	0.09
	12 Months			0.15	0.06

**Figure 3:**
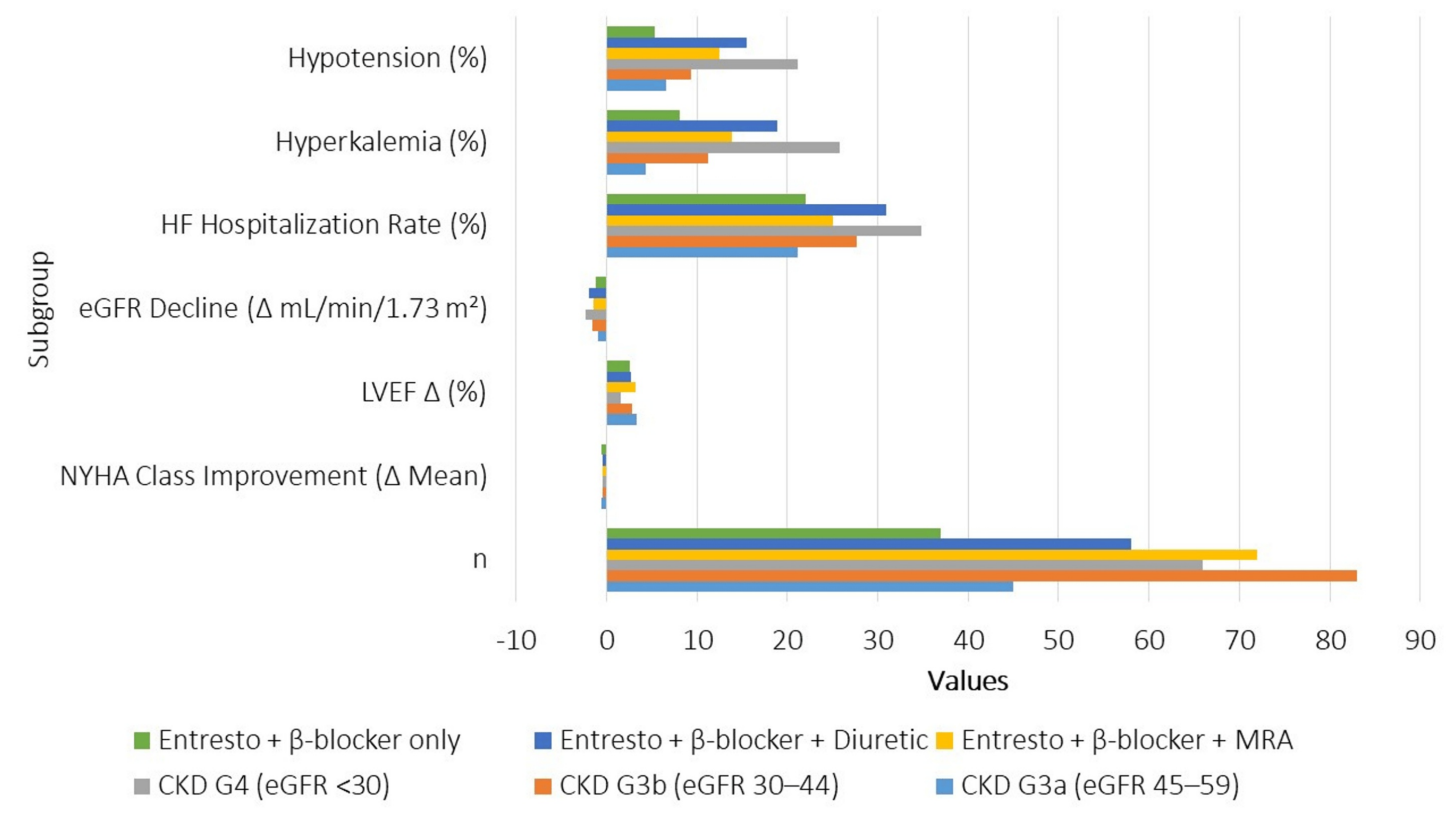
Subgroup Analysis Subgroup analysis compared clinical outcomes across CKD stages (G3a, 45-59; G3b, 30-44; and G4, <30 mL/minute/1.73 m²) and by HF medication combinations including sacubitril/valsartan with beta-blockers, mineralocorticoid receptor antagonists (MRAs), and loop diuretics. Outcomes assessed included improvement in NYHA class, changes in LVEF, decline in estimated glomerular filtration rate (eGFR), HF-related hospitalization, and the incidence of adverse events (hyperkalemia and hypotension). Only hospitalizations confirmed for HF exacerbation were included. This stratification helped identify high-risk profiles based on renal function and concurrent pharmacotherapy CKD, chronic kidney disease; HF, heart failure; LVEF, left ventricular ejection fraction

The subgroup analysis (Figure 3) yielded several compelling insights into how renal function and drug combinations influenced treatment outcomes. Patients with milder CKD (G3a) experienced the most pronounced benefits, including the greatest improvement in NYHA functional class and LVEF, along with the lowest rates of HF-related hospitalization and adverse events. In contrast, those with severe CKD (G4) showed limited cardiac gains and a more marked eGFR decline, accompanied by higher rates of hyperkalemia, hypotension, and hospitalizations, highlighting the clinical complexity and vulnerability of this subgroup. Interestingly, patients receiving sacubitril/valsartan with beta-blockers alone had the most favorable safety profile, while those also taking loop diuretics showed a steeper renal decline and higher hospitalization rates, suggesting the need for cautious diuretic use, especially after decongestion. The triple combination with MRAs yielded moderate functional benefits but a higher risk of hyperkalemia, reinforcing the importance of potassium monitoring. These findings support a more tailored, renal-function-guided approach to optimizing HF therapy in patients with coexisting CKD.

## Discussion

This 24-month observational study highlights the real-world performance of sacubitril/valsartan in patients with HFrEF and concomitant CKD. A significant and sustained improvement in NYHA functional class and a modest but statistically meaningful rise in LVEF were observed, consistent with prior reports from pivotal trials [23] and more recent real-world studies [24,25]. These functional gains likely reflect the dual mechanism of neprilysin inhibition and angiotensin receptor blockade, which enhances natriuresis, vasodilation, and cardiac remodeling reversal [26].

Our study expands upon findings from the PARADIGM-HF trial, which demonstrated improved survival and fewer hospitalizations with sacubitril/valsartan compared to enalapril [23]. However, unlike PARADIGM-HF, which excluded patients with severe renal impairment, our cohort had a substantial representation of patients with a baseline eGFR of <30 mL/minute/1.73 m². This allowed the assessment of renal safety in this vulnerable population. Although a small but significant decline in eGFR was observed, most patients remained within manageable CKD stages, and the renal trajectory remained stable in patients with optimized volume status and individualized diuretic management.

A key strength of this study is its prospective design and relatively long follow-up duration. Unlike trials that often exclude patients with multiple comorbidities, our sample reflects a high-risk, real-world population with over 57% diabetic patients and 71% hypertensive individuals. Furthermore, a modest positive correlation (r = 0.21; p = 0.004) between LVEF improvement and eGFR preservation suggests a cardiorenal interplay favorably influenced by sacubitril/valsartan. This aligns with findings from the ENVI study, which demonstrated left ventricular reverse remodeling through combined ARNI and sodium-glucose cotransporter-2 (SGLT2) inhibitor therapy [27].

Sacubitril/valsartan, a first-in-class angiotensin receptor-neprilysin inhibitor, has shown significant reductions in cardiovascular mortality and heart failure hospitalizations in HFrEF patients, as evidenced by the PARADIGM-HF trial [28]. In our study, a pie chart comparison illustrates the frequency of confirmed heart failure-related hospitalizations at baseline and at 24 months. Blue segments indicate baseline distribution, while orange segments represent data at 24 months. Over time, a modest decline was observed in patients without hospitalizations, with a corresponding increase in those experiencing one or more hospitalizations. Only admissions for confirmed heart failure exacerbations were included, excluding routine diagnostic or follow-up admissions to minimize bias. The observed shift highlights the progressive nature of heart failure, even under guideline-directed therapy, and underscores the importance of continuous monitoring and individualized management.

The lack of a comparator group limits causal inference, and the improvements observed should be interpreted as associations. Hospitalizations were adjudicated carefully; only HF-related admissions were included, and diagnostic follow-ups were excluded to minimize bias. The rising hospitalization rates over time, despite functional gains, may reflect HF progression, renal deterioration, or suboptimal long-term adherence to adjunct therapies. Importantly, we stratified the adverse event profile, hyperkalemia and hypotension, by baseline renal function, revealing that severe CKD patients were more prone to these events, necessitating close electrolyte monitoring and the careful use of potassium-sparing drugs [29].

Nevertheless, the data also reveal a progressive decline in eGFR over time, with a growing number of patients shifting into more severe CKD categories. While this could partially be attributed to the underlying trajectory of CKD, concurrent factors such as persistent neurohormonal activation or diuretic overuse may contribute. Approximately one in four patients had a baseline eGFR of <30 mL/minute/1.73 m², highlighting the real-world application of ARNI therapy in a high-risk group. Among the 20 patients with marked renal deterioration, we identified risk profiles including higher NYHA class, diabetes, vascular disease, and sustained diuretic exposure (see Appendices), the latter of which has been linked to increased mortality if not down-titrated after congestion resolves [30].

To mitigate these risks, particularly hyperkalemia and further renal impairment, several renal-protective strategies were adopted. These included individualized down-titration of mineralocorticoid receptor antagonists, dietary potassium moderation, and the suspension of nephrotoxic medications during intercurrent illness. This aligns with emerging CKD management frameworks that emphasize polypharmacy reduction and tailored medication review [31,32]. The gradual increase in confirmed heart failure-related hospitalizations, even in the context of improved LVEF and NYHA class, reinforces the chronic, progressive nature of HF and the need for close monitoring.

Study strengths and limitations

This study's key strength lies in its prospective, 24-month follow-up design, enabling the longitudinal assessment of clinical, cardiac, and renal parameters in patients with HFrEF and coexisting CKD. The integration of both echocardiographic and renal endpoints offers a comprehensive evaluation of sacubitril/valsartan's real-world utility in this high-risk cohort. All patients were initiated and monitored in a uniform clinical protocol.

Several limitations must be acknowledged. First, the absence of a comparator group limits the ability to infer causality; observed changes should be interpreted as associations rather than definitive treatment effects. Second, the single-center setting and modest sample size may restrict generalizability. Third, potential confounding factors such as adherence, dietary sodium intake, and comorbidity burden were not fully captured. Although efforts were made to minimize bias, such as excluding hospitalizations for routine follow-up or testing, the possibility of selection bias remains. Lastly, while the "real-world" nature of the cohort reflects clinical practice, this term refers to a consecutively enrolled, non-trial population managed at a tertiary cardiac care center.

## Conclusions

In this prospective cohort of HFrEF patients with moderate-to-severe CKD, sacubitril/valsartan use was associated with improvements in NYHA class and LVEF over a 24-month period, alongside a modest but statistically significant decline in renal function. These findings suggest a potential clinical benefit in functional status and cardiac performance, though causality cannot be established due to the observational nature of the study. A notable proportion of patients transitioned from NYHA Classes III-IV to I-II, and the mean LVEF improved by nearly 3%, mirroring findings in larger trials. However, over time, renal function declined progressively, with more patients entering severe CKD categories. This may reflect either the natural trajectory of comorbid HF-CKD or the impact of neurohormonal blockade, including ARNI therapy, on glomerular hemodynamics.

Although HF-related hospitalizations increased modestly, the majority of patients avoided readmission by 24 months. Adverse events such as hyperkalemia and hypotension were consistent with known safety profiles and manageable with protocol-guided dose adjustments. Future studies should incorporate control arms, stratified analyses by CKD stage, and broader comorbidity profiling to validate these findings. In the interim, our results support the cautious use of sacubitril/valsartan in patients with cardiorenal comorbidity, emphasizing the need for close monitoring and individualized therapy to balance benefits and renal safety.

## Appendices

Table 7 shows the clinical profile of patients with worsened eGFR at 24 months.

**Table 7:** Clinical Profile of Patients With Worsened eGFR at 24 Months (n = 20) Patients who did not have their diuretic therapy down-titrated despite clinical stabilization are marked, as this is associated with increased mortality risk [30] DM, diabetes mellitus; CAD/CVD, coronary artery disease/cerebrovascular disease; eGFR, estimated glomerular filtration rate; LVEF, left ventricular ejection fraction

Patient ID	Baseline eGFR (mL/minute/1.73m²)	eGFR at 24 Months	LVEF (%)	DM	CAD/CVD	Loop Diuretic (High Dose)	Diuretic Down-Titrated
1	32	22	30	Yes	CAD	Yes	No
2	28	20	32	Yes	Both	Yes	No
3	29	21	34	No	None	No	Yes
4	30	19	35	Yes	CVD	Yes	No
5	31	23	33	No	CAD	No	Yes
6	33	24	30	No	None	Yes	Yes
7	27	19	31	Yes	Both	Yes	No
8	29	21	34	Yes	CAD	No	Yes
9	30	20	33	Yes	CVD	Yes	No
10	28	22	32	No	None	Yes	No
11	26	18	29	Yes	CAD	Yes	No
12	25	17	28	Yes	CAD	Yes	No
13	31	20	35	No	CVD	No	Yes
14	30	21	36	Yes	CAD	Yes	Yes
15	28	19	34	No	None	No	Yes
16	29	20	30	Yes	Both	Yes	No
17	27	18	29	Yes	CAD	Yes	No
18	25	17	28	Yes	CVD	Yes	No
19	26	19	32	No	CAD	No	Yes
20	28	20	31	Yes	CAD	Yes	No
